# How Laboratory Experiments Can Be Exploited for Monitoring Stress in the Wild: A Bridge Between Laboratory and Daily Life

**DOI:** 10.3390/s20030838

**Published:** 2020-02-04

**Authors:** Yekta Said Can, Dilara Gokay, Dilruba Reyyan Kılıç, Deniz Ekiz, Niaz Chalabianloo, Cem Ersoy

**Affiliations:** Computer Engineering Department, Bogazici University, Bebek, 34342 Istanbul, Turkey; dilara.gokay@boun.edu.tr (D.G.); dilruba.kilic@boun.edu.tr (D.R.K.); deniz.ekiz@boun.edu.tr (D.E.); niaz.chalabianloo@boun.edu.tr (N.C.); ersoy@boun.edu.tr (C.E.)

**Keywords:** smart band, stress recognition, physiological signal processing, machine learning

## Abstract

Chronic stress leads to poor well-being, and it has effects on life quality and health. Society may have significant benefits from an automatic daily life stress detection system using unobtrusive wearable devices using physiological signals. However, the performance of these systems is not sufficiently accurate when they are used in unrestricted daily life compared to the systems tested in controlled real-life and laboratory conditions. To test our stress level detection system that preprocesses noisy physiological signals, extracts features, and applies machine learning classification techniques, we used a laboratory experiment and ecological momentary assessment based data collection with smartwatches in daily life. We investigated the effect of different labeling techniques and different training and test environments. In the laboratory environments, we had more controlled situations, and we could validate the perceived stress from self-reports. When machine learning models were trained in the laboratory instead of training them with the data coming from daily life, the accuracy of the system when tested in daily life improved significantly. The subjectivity effect coming from the self-reports in daily life could be eliminated. Our system obtained higher stress level detection accuracy results compared to most of the previous daily life studies.

## 1. Introduction

Stress, an ever-growing issue in modern societies, has become an inseparable part of people’s fast-paced daily lives. Continuously increasing workload, tight deadlines, and the resulting time pressure all contribute to the increasing stress levels. Stress is an organism’s reaction mechanism to a stressor. In a stressful state, certain control systems in the human body, such as the autonomic nervous system (ANS), act mostly unconsciously to control the responses to stress by regulating some bodily functions. This mechanism has been constantly improved throughout human evolution, to create prompt canny reactions in life-threatening situations [1]. Stress symptoms can be measured and observed in numerous ways. The sympathetic nervous system (SNS) kicks off the stress reaction, which will appear in the form of psychological, physiological, and behavioral indications [1]. The bidirectional impacts of the mind on the body and vice versa are among the major hallmarks of the autonomic nervous system (ANS), which has evolved in a way to have a direct role in human life and survival [2]. The autonomic nervous system (ANS) is divided into the sympathetic nervous system (SNS) and the parasympathetic nervous system (PNS). Most of the studies investigating the effects of chronic psychological stress on the human body have concluded that the sympathetic and parasympathetic nervous systems become over and under activated respectively, while the individual is under psychological stress. The resulting abnormal activities of the sympathetic and parasympathetic nervous systems cause physical, behavioral, and affective irregularities. In order to regulate the physiological arousal states, a balance of activity is expected between the sympathetic and parasympathetic subclasses of the autonomic nervous system (ANS). It is feasible to measure and evaluate the autonomous nervous system (ANS) function through non-invasive physiological phenomena like the electrodermal activity (EDA) and heart rate variability (HRV). For example, the high frequency (HF) component of the HRV, which is one of the frequency domain characteristics of the heart rate variability (HRV), is an indicator of the vagus nerve and parasympathetic nervous system’s (PNS) activity. In contrast, low frequency (LF) reflects the activity of the sympathetic nervous system (SNS) [3,4,5]. The final goal concerning almost all of the stress detection research is to find ways to notify the user about their stress levels and help them to manage it to avoid the social, economic, and health-related consequences.

Traditional approaches for measuring stress are taken either by a psychologist interviewing the subject or by requesting the study subject to fill in particular questionnaires designed explicitly for self-reporting. Such interviews require the constant presence of a trained psychology specialist. Requesting the subjects to fill out long lists of questionnaires and self-report diaries are the most widely adopted processes to evaluate stress. These techniques are the current gold-standard as well. However, these methods are cumbersome and entirely manual. There are also other concerns and drawbacks regarding the interviews, self-report diaries, and questionnaires due to the way that different subjects commonly behave. Since these reports are based on or influenced by personal feelings, tastes, or opinions, they are highly subjective. As an example, some people tend to respond to questions in a manner that will be viewed favorably by others [6]. Some responders may feel that sharing their private psychological feelings may put them to shame and hide their real feelings by understating them. In contradiction to that, there are numerous cases in which responses are exaggerated [7]. Gender differences also play an essential role in how men and women express and report their stress levels and affect states when confronted with various stressors [8]. However, for everyday life, the self-reports are the closest labels to the ground truth.

Any potential automated stress detection framework for daily life will be developed using a mechanism that tries to surmount the need for any intervention from a psychologist and making the whole process less incommodious. Due to the disadvantages that self-report questionnaires’ possess, research has emerged detecting regular psychophysiological signs of stress with machine learning (ML) algorithms utilizing the reliable and proven indicators such as response activities of the sympathetic nervous system, skin temperature (ST), electrodermal activity (EDA), and electrocardiogram (ECG) [9]. The purpose of adopting ML methods for raw psychophysiological objective data is to extract meaningful emotional and affective information. The raw sensor data are collected in this process and transformed into information-containing features. Some of those features would then be used while assigning affective state labels. Subjective data are also recorded by taking records of the known context and/or daily questionnaires and ecological momentary assessments (EMAs). Finally, the primary purpose of the whole process is to train machine learning algorithms to diagnose different types of behavioral and emotional states by using the EMAs, questionnaires, and the known context and test the system using the rest of the features and any feature recorded in the future. Although some of the steps described above are not necessarily required for deep learning models, the whole process for almost all of the traditional machine learning systems is almost the same [10]. In an automated stress detection scheme, these psychophysiological signals are utilized, and analysis of these signals reveals the frequency and intensity of the stress experienced by the subjects.

Preliminary studies of automated stress detection were held in the laboratory environments, and then, the research took a step into daily life since researchers realized that the stress level experienced in the laboratory is different from daily life stress [11]. However, the problems encountered in this new environment were both more complicated and intricate. Achieving precise annotations and identification of the perceived stress in the wild is a difficult task due to the high diversity in human psychological evaluation and the lack of direct observation over subject activities. Furthermore, the unrestricted movements of subjects in the wild may cause misinterpreting of stress detection by causing artifacts in the signal data. Lastly, since the medical-grade devices with cables, electrodes, and boards cannot be used in daily life due to their obtrusive nature, more pervasive and comfortable devices should be used. However, alternatives such as smart bands and smartwatches have lower data quality, and more advanced signal processing techniques should be applied to overcome this problem. Due to the issues mentioned above, the performance of daily life stress detection systems is lower than those proposed for laboratory environments. Smartwatches and smart bands are unobtrusive, easy to use without requiring specific actions, and are more suitable for daily life. They are adopted by the consumer and easily available on the market. Most of them are equipped with photoplethysmography (PPG) sensors [12]. Our solution is easily applicable to consumers due to the availability of these devices.

Given the complexity of data acquisition solely performed in the wild in addition to the advantages of the in-lab data, consequently, the question arises as to whether it is possible to combine these systems somehow and use the high accuracy from the first for the sake of the latter. One of the questions that stands out the most is the feasibility of combinations of these two systems to achieve even better results. In this study, we will represent a framework by which it is possible to design a stress detection mechanism that utilizes both of those methods. The data collected in a laboratory can be used for the long-term performance evaluation of the same system in daily life scenarios.

Our work contributes to state-of-the-art in four different aspects:

- Developing laboratory-based models to improve the performance of daily life stress detection systems;
- Comparing the performance of laboratory, daily life, and hybrid laboratory-daily life models;
- Collection of smartwatch-based EDA and HRV data coming from the laboratory and daily life (14 participants, 1003 h of physiological data with 388 ecological momentary assessments (EMAs)), with self-reports and context information;
- Investigating the effect of using context and self-report labels while training the model on system accuracy in different environments.

These contributions will provide insights for researchers into how to improve the performance of daily life stress detection systems.

The organization of the rest of the paper is as follows. In Section 2, we present the related work in stress detection and alleviation. Our proposed unobtrusive system for stress level monitoring with smart bands is described in Section 3. Data collection procedures are explained in Section 3. Experimental results and discussion are presented in Section 4. We present the conclusions of the study in Section 5.

## 2. Related Work

Most of the automatic stress detection studies in the literature were conducted either in the laboratory environments or restricted daily life settings. We can examine the studies in the literature as five different classes. The first class develops a laboratory model with known context labels and tests in the same environment. Since the stressor levels (i.e., the context participants are in) are known at any time in laboratory experiments, they could be used as the ground truth labels for machine learning (ML) models, and we called this type laboratory-to-laboratory known context (LLKC) models. The second type uses collected self-report labels in laboratory environments and tests the created model in the same environment. We call this type laboratory-to-laboratory self-report (LLSR) models. The third type is using self-report questionnaires collected in the wild and testing the model in the same environment. We named this model daily-to-daily self-report (DDSR) models. Since we could not monitor the everyday life of participants and get the ground truth all the time, a known context does not exist in daily life environments. The laboratory data could be used to enhance daily life stress detection models. If the known context labels in the laboratory are used for an ML model development, we call this fourth type laboratory-to-daily known context (LDKC) models. On the contrary, if the self-reports in the laboratory are used as labels and the developed models are tested in the wild, we name the fifth type laboratory-to-daily self-report (LDSR). Table 1 illustrates some of the studies conducted either in the laboratory, in daily life, or both. In this section, we will briefly outline some of the research for stress detection that has been conducted in the laboratory and everyday life environments by using the taxonomy developed above.

Early experimental practices in the laboratory were the first building blocks of this research field. These preliminary studies provided researchers with the idea of choosing the most proper devices, features, and machine learning algorithms that can be used later in everyday life settings. In a trade-off between unobtrusiveness and accurate signals, researchers chose the types of devices with different sensing technologies based on the requirements of their study. Several researchers demonstrated that wearable devices equipped with PPG sensors added more comfort and convenience compared to ECG devices for HRV measurements [28,29,30]. Nevertheless, single-lead electrocardiogram (ECG) devices are becoming more compact, easy to wear, and commercially available over time. In a recent study by Billeci et al., single-lead ECG was used to study the autonomic nervous system response through monitoring the heart rate and HRV features [31]. In [14], Zubair et al. developed a smart wristband that has EDA, Bluetooth, and accelerometer sensors to detect the stress values of the test subjects. In their designed scheme, EDA and the accelerometer were used together to enhance the detection accuracy. Their experiments resulted in 91% accuracy for two-class stressed-relaxed classification. Castaldo et al. administered a study in a laboratory environment to investigate the effects of ultra-short HRV (heart rate variability) with a two minute duration on stress detection accuracy [15]. By applying the support vector machine (SVM) to HRV features, they could score 70% accuracy on their binary classification problem. Although a 70% accuracy seems low compared to other studies in the same year, they speculated that this could be due to the stress induction methods used and ultra-short-term HRV features. Tivatansakul et al. used HRV features inferred from the ECG signals and facial expressions to recognize negative emotions in the laboratory environment. Subjects were exposed to the International Affective Picture System (IAPS) set of disturbing images for four minutes. After applying the SVM classifier on the extracted features, classification accuracy for the negative emotion detection was 83% [20]. All of the in-lab implemented cases mentioned above used only one biosignal, either EDA or ECG, in their proposed stress detection mechanism. Other studies practiced multimodality by employing multiple biosignals to achieve even higher accuracy levels. In a recent stress detection and alleviation research work, Akmandor et al. [21] recorded EDA, ECG, blood pressure, blood oximeter, and respiration rates of the participants. In order to induce stress, memory games and IAPS were used, and for the alleviation part, they used various stress mitigation techniques such as classical music and micro-meditation. Their classification accuracy using SVM and kNN for their binary class problem was 95.8% [21]. Researchers used a combination of medical-grade devices with the highest possible accuracies in the laboratory settings. For instance, there are cases in which electroencephalogram (EEG) signals were used for stress monitoring [17]. Implementing such a setting in the wild is practically impossible due to the obtrusive nature of the EEG devices and the lack of daily-life suitable wearable devices with EEG capability.

In other cases, researchers decided to mitigate the trade-off between the accuracy and unobtrusiveness and conducted their studies either in the lab or in the wild using unobtrusive wearable devices. The choice of device type (i.e., whether to use medical-grade obtrusive devices with cables, electrodes, boards, or not) is important because it determines the applicability of the system to daily life, which is the main and final goal of all the stress detection studies. In some of the studies, the data were collected in the wild and trained and tested in these environments [22,23,24,25] (DDSR model type in the taxonomy). Ciman et al. [22] detected stress by analyzing smartphone usage behaviors. In the controlled laboratory experiment, by developing an Android app with search and typing tasks, participants’ tap, swipe, scroll, and text input gestures were recorded. These gestures were expected to induce stress on the participants. They obtained approximately 80% stress detection classification accuracy with SVM, NN, kNN, and decision tree classifiers. The physical activities of the user, the light values of the screen, and the events related to the mobile phone screen were recorded in the wild experiment. The classification accuracy was about 70% with the same classifiers. Vildjounaite et al. [24] used mobile phone usage data and implemented MPM (Maximum Posterior Marginal) and HMM (Hidden Markov Model) for stress recognition. They experimented with 28 subjects for four days and obtained a maximum of 68% accuracy on their semi-personal data. Mishra et al. [25] detected stress by analyzing heart activity signals captured with a Moto360 smartwatch. The data were collected from 23 subjects for three days. They also added the activity type to physiological features to increase the accuracy of the system. Random forest (RF) was used for the classification of stressed and relaxed sessions. They increased their F1 score from 0.50 to 0.76 by adding the activity-related contextual information. A recent daily life study was carried out with 1002 participants using unobtrusive wearables [32]. They extracted heart rate variability features and employed the RF classifier. They could only achieve a 0.43 F1 score (with three classes) and listed the additional difficulties of working in the wild while explaining the reasons for the relatively low performance [32]. The performance of daily life stress monitoring systems is lower than studies conducted in controlled environments due to the mentioned issues such as low-quality physiological signals with artifacts and the difficulty of collecting the ground truth in [33,34] (see Table 1).

The data collected in the experiments conducted in the laboratory could be used for developing models in the wild to improve the reported low accuracies. There is a limited number of studies using laboratory data for creating models with higher performance in daily life. Li et al. [27] built their model in the lab, verified its performance in the lab with different participants, and later verified it in the field test again with new subjects. They used mental arithmetic tasks to induce stress and seven-point scale Likert self-reports as labels in the laboratory (LDSR model type). They evaluated their system performance by using the Elastic-net regression technique. The correlation results of predicted labels and ground truth self-reports were 0.72 and 0.56 for laboratory and daily-life settings, respectively. In another study, Gjoreski et al. proposed a continuous stress detection scheme, which consisted of a base detection mechanism solely using the laboratory data, an activity recognizer for identifying the contextual information, and a real-life stress detection mechanism that utilized the results from the laboratory detector and the context information for real-life stress detection [26]. While training the model, they used only the context label in the laboratory environment (LDKC-type of model). They did not collect self-reports during these tests. The classification accuracy of their stress detection system without using context information was 76% [26] (they increased it to 92% by adding the activity type). Since different methods that were mentioned in our taxonomy were tried in different datasets created for each particular paper in the literature (see Table 1), one could not infer the success of a method over others. Therefore, there is clearly a need for comparing the different proposed techniques for stress detection in the laboratory and daily life settings with unobtrusive devices.

## 3. Methodology

### 3.1. Laboratory Data Collection

We collected controlled laboratory data from the participants during a psychological experiment using our implementation of the Trier Social Test (TSST) [35]. The social test has been proven to be an academically effective way of inducing stress after the baseline condition we created at the beginning of the experiment. This experiment was conducted on 14 different participants who were university students aged between 20 and 25. There were nine male and five female participants.

The experiment, which took approximately 1 hour, had the following steps:

1. Setup
2. Pre-stress measurements (baseline)
3. The TSST (Trier Social Stress Test) (inducing stress)
4. Post-stress recovery measurements (recovery)

The communication language between interviewers and the participants was Turkish. Please note that the mother tongue of all participants was Turkish. In addition to that, they knew English as a foreign language. This circumstance affected stress induction.

#### 3.1.1. Setup

The setup was done as follows;

1. Preparation of experiment areas: The camera should be set. Empatica E4 should be ready.
2. Interviewers should keep eye contact with the participant. Their gestures and facial expressions should be neutral.
3. The participant is informed about the procedure and then signs the consent form.
4. The participant wears the smart band (Empatica E4).
5. The participant is asked to turn off his/her phone in order to eliminate distraction.

#### 3.1.2. Pre-Stress Measurements

**Figure 1:**
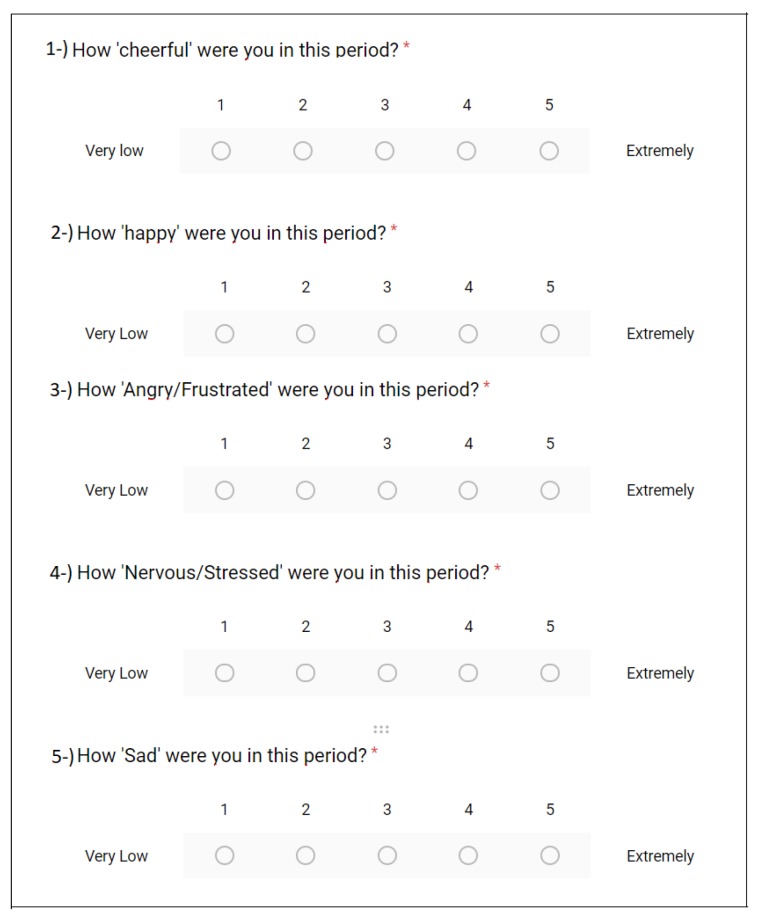
The Perceived Stress Scale (PSS)-5 questionnaire used in the experiment.

Before the experiment, the following procedure was applied;

1. The participant filled out the Perceived Stress Scale (PSS) with 14 questions.
2. The participant was told to stay in the waiting area and get rest for 10 min. Reading materials such as magazines with emotionally-neutral contents (home and garden, car magazines) were presented to the participant for this period.
3. The participant filled out the PSS-5 (ambulatory PSS) form. This questionnaire was first created by Cohen et al. [36] and used for measuring the perceived stress in ambulatory settings in [37] (see Figure 1).

#### 3.1.3. The TSST

**Figure 2:**
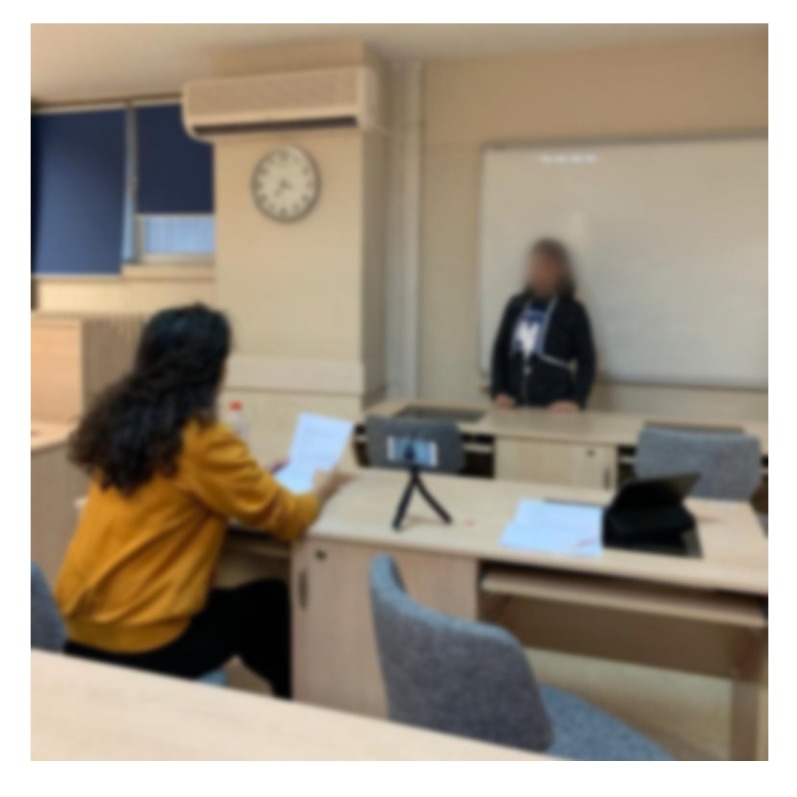
An example scene from the TSST phase in our experiment. The participant is presented at this moment in front of the neutral experimenter.

Our implementation of the TSST is described as follows:

1. The participant was directed to the interview area.
2. TSST speech preparation period: the following text was read to the participant: “This is the speech preparation portion of the task; you are expected to prepare a five-minute speech describing why you study [name of the degree that the participant studies/studied] and why you would be a good candidate for your ideal job. Your speech will be videotaped and reviewed by the psychologists that we conduct the research with. You have five minutes to prepare and your time begins now.”
3. The participant prepared his/her speech. There should be a digital timer in the room set to five minutes. Interviewers should leave the room.
4. The following text was read to the participant at the end of the speech preparation period: “This is the speech portion of the task. You should speak for the entire five-minute time period. Your time begins now”. Interviewers should start the recording of the camera.
5. TSST speech performance period: If the participant stopped during this period, interviewers allowed him/her to stay silent for around 20 s and then prompted: “You still have time remaining.”
6. After the first 2 min of the speech period, the participants were interrupted and asked to continue their speech in English by telling them: “Could you continue in English from now on, please?”
7. At the end of 2.5 min, if the participant did not attempt to reply to both questions, interviewers prompted the participant to answer the other question.
8. At the end of the speech performance period, the communication between interviewers and the participant resumed in Turkish. Interviewers reset the timer to 5 min and read the following to the participant: “During the final five-minute math portion of this task, you will be asked to subtract 13 from 1022 sequentially. You will verbally report your answers aloud, and be asked to start over from 1022 if a mistake is made. Your time begins now.” If the participant makes any mistake, the interviewer says the following: “That is incorrect, please start over from 1022.” (Figure 2)
9. Participant filled out the PSS-5 questionnaire.

#### 3.1.4. Post-Stress Recovery Measurements

In order to alleviate the stress response, we applied a biofeedback based intervention, which was the built-in breathing application of Apple Watch [38]. The procedure was applied as follows:

1. Participants were directed to the couch as a relaxing place.
2. Participants wore an Apple Watch given to him/her at this stage, followed the breathing exercise built in the Apple Watch for a minute and then followed a mindfulness video, for the remaining four minutes, on a comfortable couch, sitting or lying as the participant preferred.
3. Interviewers should leave the room after giving the Apple Watch.
4. At the end of the five minute long recovery period, interviewers returned to the room, and the participant filled out the PSS-5 questionnaire.

### 3.2. Daily Life Data Collection and Ecological Momentary Assessment

After the controlled room experiments were finished, we gave the Empatica E4 devices to all participants. They were told to wear the Empatica E4 devices for twelve hours per day, between 9 a.m. and 9 p.m., for seven days. These days were not necessarily consecutive. We applied the EMA to collect information about the subject’s stress level [39]. EMA involved the repeated sampling of subjects’ current behaviors and experiences in real time, in subjects’ natural environments [39]. EMA aims to minimize recall bias, maximize ecological validity, and allow the study of micro processes that influence behavior in real-world contexts. We implemented an online version of the PSS-5 questionnaire. For each three hour session, we asked the participant to fill in the EMA. In order to make sure the collection of self-reports, we sent them e-mails over seven days at the end of each three hour session when they were wearing the wristband. In other words, the participants were reminded to fill in the PSS-5 at 12 p.m., 3 p.m., 6 p.m., and 9 p.m. over seven days. The EMA was delivered to the participants through a survey app. The app was available on both desktop and mobile browsers. The link to the EMA was delivered to the participants through e-mail. Each e-mail that was sent in order to remind participants to fill in the EMA contained the link to the EMA. This questionnaire is strongly correlated with PSS-14 and appropriate for ambulatory settings. In total, we obtained 1003 h of physiological data and 388 EMAs (including 14 h of physiological data and 56 EMAs collected in the lab). There were some sessions with missing EMAs (60 EMAs in total), and we disregarded their physiological data.

The procedure of the methodology used in this study was approved by the Institutional Review Board for Research with Human Subjects of Boğaziçi University with Approval Number 2018/16. Prior to the data acquisition, each participant received a consent form that explained the experimental procedure and its benefits and implications to both society and the subject. The procedure was also explained verbally to the subject. All of the data were stored anonymously.

### 3.3. Stress Recognition Framework

**Figure 3:**
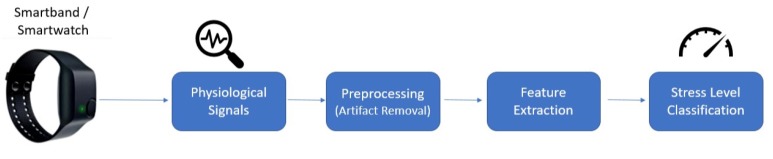
A high level block diagram of the stress level detection system with the Empatica E4 wristband.

In order to propose an unobtrusive stress detector suitable for everyday use, we used an Empatica E4 [40] comfortable wristband, which has more than 48 h of battery life and is equipped with a variety of sensors such as the three-dimensional accelerometer (32 Hz), the continuous heart rate monitoring unit based on the photoplethysmography (PPG), the skin temperature (4 Hz), and the EDA sensors (4 Hz). The major difference between the wristbands and contact sensors used in hospital settings is the vulnerability to the motion artifacts due to their design and attachment to the body. A daily life suitable and comfortable stress detection system should consider these artifacts. Thus, it should have a preprocessing unit that detects and removes the artifacts due to contact loss and motion. In daily life, the activity of the individual is important; for example, HRV and EDA can change due to high-intensity activity in short periods. Therefore a single sensor-based system can fail. Hence, a stress recognition system suitable for daily life should be multi-modal in terms of the collected data. We used robust preprocessing and feature extraction modules from our previous work [18] for this purpose (see Figure 3).

#### 3.3.1. Preprocessing

The physiological signals coming from different sensors were segmented into non-overlapping time windows. We selected the window size as two minutes since it was reported that the duration of stress stimulation and recovery processes was approximately a few minutes and these segment sizes could capture such processes [41]. IBI (Interbeat Interval) and EDA signals were sent to the artifact detection units. Therefore, the response time of our system was approximately two minutes.

The artifact detection and removal unit for the heart rate signal applied an artifact detection percentage threshold between the time of the successive R to R interval recordings. The threshold was selected as 20% [42]. After the removal of the artifacts, they were replaced with a cubic spline interpolation function, as applied in Kubios [43]. The interpolation method was selected for applying time and sample constraints on the remaining data since it achieved better results [18]. The windows containing more than 10% of RR interval artifacts were removed. This unit was developed in our previous work [18].

For the EDA artifact detection unit, we used the toolbox developed by Taylor et al. [44]. It uses the SVM classifier and detects the artifacts in the EDA signal with 95% accuracy by analyzing skin temperature, accelerometer, and skin conductance signals. We added a batch processing feature to this toolbox and removed the detected artifacts. Then, the resulting signal was transferred to the EDA feature extraction unit.

#### 3.3.2. Feature Extraction

To create a multi-modal stress recognition framework, we extracted state-of-the-art features from the EDA and RR intervals to create a feature vector. In this section, we describe the feature extraction methodology for each of the physiological signals. We decomposed the EDA signal into phasic and tonic components using the convex optimization-based EDAcvx tool [45]. EDAcvx also cleans the noise in the EDA signal. We extracted seven features from both the phasic and tonic components of the EDA signal. Percentiles are very handy for exploring the distribution of number sets using various EDA graphs. These following EDA features were selected from the literature [13,26].

1. Mean value
2. Standard deviation
3. Number of peaks
4. Number of strong peaks
5. Twentieth percentile
6. Eightieth percentile
7. Quartile deviation

We extracted 13 heart rate variability (HRV) time and frequency domain features from RR intervals. These features were commonly used in the previous works [11,26,43,46,47]. In order to compute frequency domain features, we re-sampled RR intervals at 4 Hz [48] and applied fast Fourier transform (FFT). The computed HRV features are shown below:

1. Mean value of the inter-beat (RR) intervals
2. Standard deviation of the inter-beat interval
3. Root mean square of the successive difference of the RR intervals.
4. Percentage of the number of the successive RR intervals varying more than 50 ms from the previous interval
5. Total number of RR intervals divided by the height of the histogram of all RR intervals measured on a scale with bins of 1/128 s
6. Triangular interpolation of RR interval histogram
7. Power in the low-frequency band (0.04–0.15 Hz)
8. Power in the high-frequency band (0.15–0.4 Hz)
9. Ratio of LF to HF.
10. Prevalent low-frequency oscillation of the heart rate
11. Prevalent high-frequency oscillation of the heart rate
12. Power in the very low-frequency band (0.00–0.04 Hz)
13. Related standard deviation of successive RR interval differences

#### 3.3.3. Feature Selection

**Figure 4:**
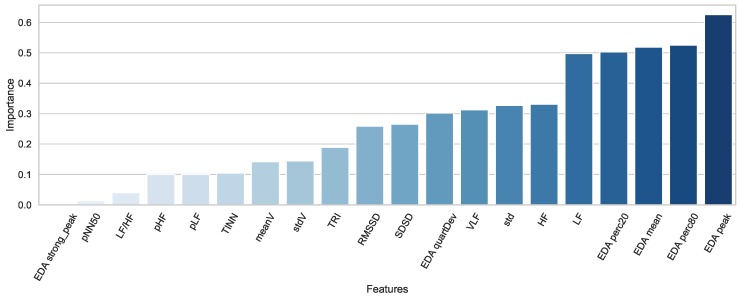
Features listed in order of importance based on correlation-based feature selection for the DDSR model. EDA peaks are the feature that has the highest importance, whereas the EDA strong peak has the lowest.

We applied correlation-based feature (CBF) [49] selection using the Weka [50] tool. The importance of the features is shown in Figure 4. Since our goal was to develop a stress detection model that works in daily life settings, we conducted experiments with the 1 to 20 best features for the DDSR model. We achieved the best results with ten features for HRV + EDA, five features for HRV, and five features for EDA. We applied the classifiers to the selected features.

#### 3.3.4. Preparation of the Data for ML Algorithms

Although we had evenly distributed data in terms of known context labels in the laboratory, we had a class imbalance problem in the self-reported ground truth labels obtained from the daily life and laboratory. In the laboratory, 71.5% of the data was relaxed and 28.5% of data was stressed. In addition, 73% of the daily life data was relaxed, and the remaining 27% was stressed. We overcame this problem by randomly undersampling the extra samples of the majority class, which is the most commonly used procedure for imbalanced datasets [51]. We further applied normalization on features to prevent overfitting. Lastly, we converted the numeric labels to the nominal type as an input to the Weka toolkit classification algorithms.

#### 3.3.5. Forming ML Models

**Figure 5:**
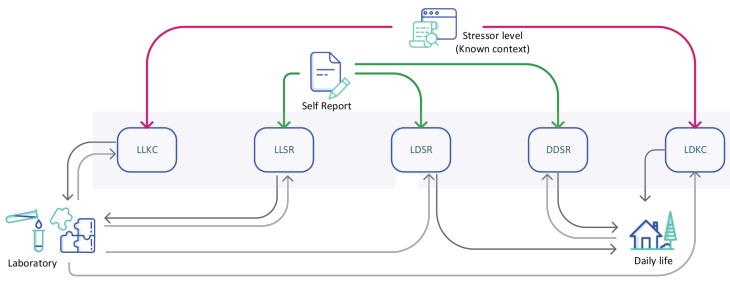
We developed five different stress level classification models with varying ground truth labels and training-test environments. Red and green arrows indicate the ground truth type used. The incoming black arrow shows the training environment, the an outgoing black arrow shows the testing environment.

We developed five different models that used different ground truth types and 248 training and test environment combinations (see Figure 5). The data were divided into two minute windows in the preprocessing part. The label of the window extracted from the time was added to the feature vector. The label could be the stressor level of the session or the perceived stress level obtained from the participants. Features extracted from all windows belonging to a session were averaged, and the label was appended to this feature vector. Data coming from all participants were merged and randomly listed. We then formed one general model from our dataset. In other words, separate models for all participants were not formed. In order to evaluate the performance of the classifiers, we applied 10-fold cross-validation by partitioning the original sample into a training set to train the model and a test set to evaluate it, then we changed the test and training until each partition was used for the test set. We further created separate training (80%) and test sets (20%) and evaluated the results to compare with 10-fold cross-validation results. In the 10-fold results, the standard deviations of the folds are provided in parentheses.

#### 3.3.6. Classification Algorithms

We used five different machine learning classifiers for recognition of stress events. These classifiers were the ones mostly used and that best performed in the literature [26,42], namely multi-layer perceptron (MLP), random forest (RF), k-nearest neighbor (kNN), support vector machine (SVM), and logistic regression (LR). For the LR, the output probability was divided into two different classes, which were above and below 0.5 [52]. We used the Weka Machine Learning Software [53] for the classification section of our proposed system.

In order to select the best parameter set for the MLP, we experimented with the different numbers of hidden layers (1, 2, and 3) and units (from 1 to 20); one outperformed others, which had two hidden layers, and each layer had five units. We experimented with N for the kNN. After parameter tuning, the best N was selected as 3. We applied the radial basis kernel (RBF) and linear kernel for the SVM. We selected RBF for SVM because it outperformed the linear kernel. For the RF, we selected the number of trees as 100.

## 4. Experimental Results and Discussion

We divided our tests into two groups: laboratory and in the wild experiments. We examined the results in the following two sections.

### 4.1. Laboratory Experiments

In this experiment, our aim was to differentiate between the stress and baseline states. In this section, we investigate the performance of our stress detection scheme in two different manners. The first one was using the known context as the ground truth. We further provide these labels as classes to the ML algorithm. The second way was to use the perceived stress levels collected from self-reports as the ground truth. In order to measure the perceived stress levels, we collected PSS-5. For these five questions, positive emotions (happy and cheerful) were evaluated inversely. In other words, if a participant stated six: extremely happy from 1–6, it was evaluated as one because happiness and cheerfulness are inversely proportional to stress levels. On the other hand, anger, sadness, and frustration were evaluated proportionally when calculating the score (see Equation (1)).

(1) $$PercStress=\left(7-H_{i}\right)+\left(7-C_{i}\right)+A_{i}+S_{i}+F_{i}$$

where $H_{i}$: happiness score, $C_{i}$: cheerfulness score, $A_{i}$: anger score, $S_{i}$: sadness score, and $F_{i}$: frustration score. Individual scores on the PSS can range from zero to 30 with higher scores indicating higher perceived stress. Scores ranging from 0–15 would be considered low stress, and scores ranging from 15–30 would be considered high perceived stress. The division was made by adapting the three class division of the PSS-14 class [54]. The performance of our system is presented in Table 2 and Table 3.

We successfully differentiated stress with baseline states, as seen in Table 2 (with a maximum of 94.4% accuracy). Perceived stress level detection classification performance is always higher than physiological stress level detection in the known context because participants may experience different stress levels than the expected level of the context. Some participants may experience lower stress in the TSST while preparing the presentation, presenting in a foreign language, or counting tasks. This proves that the choice of using the ground truth as known context labels or perceived stress labels has a significant influence on the performance of the system. The combination of the two physiological signals always achieves higher accuracy than the single modality with minimum accuracy. However, it does not give the maximum accuracy in all conditions. We could state that multi-modality has an observable effect on some conditions. Overall, we successfully differentiated between baseline and stress states in the laboratory environment.

### 4.2. Testing the Models in the Wild

As mentioned, we had two types of class labels in the laboratory environment stress detection experiment: PSS-5 self-reports and the known context (the stressor level). On the other hand, in the wild, we had only self-reports of individuals, which was among the reasons why daily life stress detection performances were low [34], and there is still a room for improvement in daily life stress detection research.

We used both laboratory ground truth labels separately while developing machine learning models for laboratory environments and testing in daily life. The LDSR model was trained with the laboratory self-report labels, whereas the LDKC model was trained with the laboratory known context labels. We expected that laboratory self-report based labeling would have higher performance than laboratory context-based labeling because we had only the perceived stress labels (questionnaires) in the wild, which were more coherent with the laboratory self-reports. Furthermore, participants might experience different perceived stress than the known context implied, which reduced the performance of the LDKC models. We further developed a DDSR model that was solely trained and tested with daily life data. We compare these three models in Table 4, Table 5, Table 6 and Table 7. As expected, the LDSR model had the best performance, whereas the DDSR model had the lowest performance. In the laboratory, collected self-reports were more reliable because the environment was controlled. The noise coming from the daily life environment (i.e., forgetting stressful events in a session, unrestricted movements) decreased the performance of DDSR models when compared to LDSR models, which had more clear training data and labels. We could state that collecting laboratory data and training a stress level detection model with that data improved the stress level detection performance in the wild. Furthermore, choosing the self-report label resulted in better performance when compared to that with the known context labels. As far as the performance of modalities is concerned, we achieved the best results with the HRV signal. In most of the daily life cases (15/20 tests), a combination of the signals achieved better results than single modalities alone. In the remaining tests, negative correlations between the selected best features from different modalities could decrease the performance of the system when modalities were combined. In daily life, RF and SVM achieved the best performances, which aligned with the recent literature [26]. Especially with the EDA signal, RF always outperformed other classifiers in daily life tests. Lastly, when the contribution of features were examined (see Figure 4), the best ones included five EDA and five HRV features, which also showed that the combination of these modalities was important.

## 5. Conclusions

Since stress detection systems have lower accuracies in the wild when compared to laboratory environments, there is a need to develop new techniques to improve their performance. In this study, we examined the effect of developing ML models in different environments and with varying ground truth labels. To the best of our knowledge, this was the first work to examine all possible combinations of perceived stress measurements for daily life and laboratory settings along with different ground truth labels. We used EDA, HRV, ST, and ACC signals (ST and ACC were used for artifact detection and removal) in our unobtrusive stress detection system. We first trained and tested our system in the laboratory environment. We obtained a maximum of 94.4% accuracy with HR, 86.70% with EDA, and 92.30% with HRV + EDA, which showed that our system detected the stress levels in the laboratory successfully. These results were aligned with the literature [14]. Choosing the ground truth as self-reports while training the ML model always achieved higher accuracies than using the known context labels, which could be explained by the fact that stressor levels (i.e., known context) might not represent the perceived stress of participants. We further took a step out to daily life environments and tried the DDSR model, which achieved 68.30% accuracy with HRV, 63.60% accuracy with EDA, and 71.40% accuracy with multimodal HRV + EDA. Then, we applied the model trained in the laboratory with self-reports and showed that the performance increased with all types of physiological signals for daily life stress recognition (7% increase for HRV, 14.8% increase for EDA, 2.8% increase for HRV + EDA). We also investigated that the mean accuracy was enhanced with the LDKC model for the HRV + EDA- and EDA-based stress recognition frameworks. The classification performance of the proposed system changed significantly based on the event labeling methodology. Models trained in the laboratory for daily life stress detection outperformed the ones trained with daily life data. We achieved the best results (73.81%) in the LDSR model, where the daily life stress detection system was trained in the laboratory environment with self-report labels. This demonstrated that we could increase the accuracy of the system by training the model in the laboratory with the same kind of ground truth since we also had self-reports as the ground truth in the wild. We also showed that multi-modal sensing provided a more robust framework for all types of session labeling approaches. The performance of the LDKC model was better than the DDSR model and worse than that of the LDSR in the multimodal framework. On the other hand, the accuracies of the LDKC model were lower than both those of the LDSR and DDSR results when only a single modality was used. We could infer that the DDSR method suffered from relatively noisy training labels and data when compared to LDSR, where training data were obtained from the controlled laboratory environment. Using different types of labels in training and testing (known context labels in the laboratory for training and self-reports in the wild for testing) might be responsible for the low performance of LDKC models. RF and SVM classifiers outperformed other classifiers, and these results were aligned with the daily life stress recognition studies mentioned in the Related Work Section. Feature and modality selection is vital for achieving better performances. We selected the best ten features; five of them were from EDA, and five of them were from HRV, which also suggested that a multi-modality approach was crucial for daily life stress detection. In most of the test cases (15/20), the combination of modalities increased the performance of the system. In the remaining tests, anticorrelations between the features of different modalities might be the cause of lower accuracies. There is still room for improvement for daily life stress recognition. Moreover, our study was not without limitations. In order to generalize the conclusions, additional studies based on larger heterogeneous sample groups are needed. As future works, we plan to develop personalized perceived stress models to overcome the subjectivity problem of self-reports. We will try to exploit baseline surveys and daily session-based questionnaires of individuals to prevent the bias caused by subjective self-reports.

## Figures and Tables

**Table 1:** Stress detection experiments in laboratory and daily life settings. EDA, electrodermal activity; IAPS, International Affective Picture System; PPG, photoplethysmography; ACC, Accelerometer; MIST, The Montreal Imaging Stress Task; SCWT, Stroop Color and Word Test; TSST, Trier Social Stress Test; BVP, Blood Volume Pressure; RR, R to R interval.

Article	Stress Signal	Stress Test	Unobtrusive	Model Type					Accuracy	
				LLKC	LLSR	DDSR	LDKC	LDSR	Lab	Daily Life
[13] (2009)	EDA, ECG, ACC, Respiration	MIST	✗	✓	✗	✗	✗	✗	82.8%	-
[14] (2015)	EDA, Bluetooth, ACC	Mixed	✗	✓	✗	✗	✗	✗	91%	-
[15] (2017)	ECG	SCWT	✗	✓	✗	✗	✗	✗	70%	-
[16] (2016)	PPG, EDA, Respiration, Thermal Camera	Lie Detection	✗	✓	✗	✗	✗	✗	73%	-
[17] (2016)	EEG	Arithmetic Task	✗	✓	✗	✗	✗	✗	89%	-
Our Previous Work [18] (2019)	PPG, EDA	Programming Contest	✓	✓	✓	✗	✗	✗	97.92%	-
[19] (2015)	EDA, PPG	TSST	✗	✓	✗	✗	✗	✗	80%	-
[20] (2015)	ECG, Facial recognition	IAPS	✗	✗	✓	✗	✗	✗	83%	-
[21] (2017)	ECG, GSR, Blood Oximeter, Blood Pressure, Respiration	Ice Test, IAPS	✗	✓	✗	✗	✗	✗	95.8%	-
[22] (2016)	Mobile App Usage Pattern, Light, Physical Activity	Daily Life	✓	✗	✓	✓	✗	✗	80%	70%
[23] (2015)	ECG + Respiratory + Accelerometer	Daily Life	✗	✗	✓	✗	✗	✓	90%	72%
[24] (2018)	Usage Data for Different Application Categories	Daily Life	✓	✗	✗	✓	✗	✗	-	68%
[25] (2018)	HR (Heart Rate)-ACC	Daily Life	✓	✗	✗	✓	✗	✗	-	0.76 precision
[26] (2017)	BVP, EDA, Skin Temperature, RR	Daily Life	✓	✓	✗	✗	✓	✗	83%	76%
[27] (2018)	PPG	Daily Life, Arithmetic Tasks	✓	✗	✓	✗	✗	✓	0.7 correlation	0.56 correlation
Our Work	PPG, EDA	TSST, Daily Life	✓	✓	✓	✓	✓	✓	94.40%	73%

LLKC: Laboratory-to-laboratory known context. LLSR: Laboratory-to-laboratory self-report. DDSR: Daily-to-daily self-report. LDKC: Laboratory-to-daily known context. LDSR: Laboratory-to-daily self-report.

**Table 2:** Stress detection accuracies with different ML algorithms: 2 class classification. On the left side, stress recognition results, which only use self-reports as the ground truth labels are presented. On the right side, known context information is used for the ground truth label. LLKC stands for laboratory-to-laboratory known context, whereas LLSR stands for laboratory-to-laboratory self-report. Ten-fold cross-validation is used. Standard deviations are shown in parenthesis. HRV, heart rate variability.

Algorithm	LLSR			LLKC		
	HR	EDA	HRV + EDA	HR	EDA	HRV + EDA
MLP	83.30 (3.04)	77.30 (8.56)	87.20 (1.19)	62.90 (1.89)	**76.20** (9.96)	**82.90** (1.52)
RF	83.30 (6.10)	**86.70** (6.78)	84.90 (2.98)	57.80 (0.14)	66.70 (9.94)	80.39 (4.22)
kNN	**94.40** (1.79)	86.40 (8.58)	89.70 (6.34)	68.6 (5.45)	73.80 (14.65)	77.10 (5.69)
Logistic Regression	**94.40** (4.76)	72.70 (7.89)	89.70 (6.28)	68.60 (3.12)	**76.20** (13.3)	80.00 (2.01)
SVM	88.90 (6.28)	77.30 (6.10)	**92.30** (5.18)	**74.30** (4.13)	73.80 (9.77)	77.10 (3.49)

**Table 3:** Stress detection accuracies with different ML algorithms: 2 class classification. On the left side, stress recognition results, which only use self-reports as the ground truth labels are presented. On the right side, known context information is used for the ground truth label. Separate training (80%) and test sets (20%) are used. LLKC stands for laboratory-to-laboratory known context, whereas LLSR stands for laboratory-to-laboratory self-report.

Algorithm	LLSR			LLKC		
	HR	EDA	HRV + EDA	HRV	EDA	HRV + EDA
MLP	92.59	84.25	94.20	69.90	**78.20**	**86.66**
RF	**93.60**	91.20	91.40	58.60	68.80	82.21
kNN	91.20	**94.00**	**95.60**	67.20	68.80	78.32
Logistic Regression	65.74	66.66	73.14	70.89	**78.20**	79.36
SVM	77.77	84.25	90.74	**75.40**	74.40	81.10

**Table 4:** The classification accuracy using the combination of two modalities and a single modality along with the DDSR (Daily-to-Daily-Self-Report) technique was provided. 10-fold cross validation is used. Standard deviations are shown in parenthesis.

Algorithm	Accuracy		
	Combined	HRV	EDA
MLP	63.50 (8.25)	**68.30 (9.66)**	56.80 (8.89)
RF	61.90 (12.94)	65.10 (14.47)	**63.60 (11.79)**
kNN	65.90 (10.97)	64.30 (15.01)	61.40 (11.26)
Logistic Regression	70.60 (16.33)	**68.30 (8.75)**	59.30 (10.85)
SVM	**71.40** (7.03)	67.50 (8.73)	62.10 (1.53)

**Table 5:** The classification accuracy using the combination of two modalities and a single modality along with the DDSR (daily-to-daily self-report) technique are provided. Separate training (80%) and test sets (20%) are used.

Algorithm	Accuracy		
	Combined	HRV	EDA
MLP	68.00	57.30	57.30
RF	52.00	66.30	64.00
kNN	60.00	65.70	56.00
Logistic Regression	64.00	65.40	58.30
SVM	**68.00**	58.20	58.20

**Table 6:** The classification accuracy using the combination of two modalities and a single modality along with the LDKC (lab-to-daily known context) technique are provided.

Algorithm	Accuracy		
	Combined	HRV	EDA
MLP	64.73	34.43	35.26
RF	68.87	34.85	68.04
kNN	70.53	57.67	65.14
Logistic Regression	62.65	39.04	52.28
SVM	**71.78**	44.39	42.32

**Table 7:** The classification accuracy using the combination of two modalities and a single modality along with the LDSR (lab-to-daily self-report) technique are provided.

Algorithm	Accuracy		
	Combined	HRV	EDA
MLP	72.20	63.41	70.95
RF	**74.61**	**71.78**	72.61
kNN	72.20	71.37	**73.02**
Logistic Regression	73.81	71.78	71.78
SVM	73.44	73.44	72.61
